# A Pilot Study on the Effects of Exercise Training on Cardiorespiratory Performance, Quality of Life, and Immunologic Variables in Long COVID

**DOI:** 10.3390/jcm13185590

**Published:** 2024-09-20

**Authors:** Asghar Abbasi, Chiara Gattoni, Michelina Iacovino, Carrie Ferguson, Jacqueline Tosolini, Ashrita Singh, Kyaw Khaing Soe, Janos Porszasz, Charles Lanks, Harry B. Rossiter, Richard Casaburi, William W. Stringer

**Affiliations:** The Lundquist Institute for Biomedical Innovation at Harbor-UCLA Medical Center, Torrance, CA 90502, USA; asghar.abbasi@lundquist.org (A.A.); chiara.gattoni@lundquist.org (C.G.); miacovino@lundquist.org (M.I.); carrie.ferguson@lundquist.org (C.F.); jackietosolini@gmail.com (J.T.); singh.ashrita.04@gmail.com (A.S.); jporszasz@gmail.com (J.P.); clanks@dhs.lacounty.gov (C.L.); hrossiter@ucla.edu (H.B.R.); casaburi@ucla.edu (R.C.)

**Keywords:** long COVID, exercise rehabilitation, immune cell subsets, inflammation, cardiopulmonary exercise testing

## Abstract

**Objectives:** Fatigue is a prominent feature of long COVID (LC) and may be related to several pathophysiologic mechanisms, including immune hyperstimulation. Aerobic endurance exercise training may be a useful therapy, with appropriate attention to preventing post-exertional malaise. **Methods:** Fourteen participants completed a pilot study of aerobic exercise training (twenty 1.5 h sessions of over 10 weeks). Cardiorespiratory fitness, 6 min walk distance, quality of life, symptoms, 7-day physical activity, immunophenotype, and inflammatory biomarkers were measured before and after exercise training. **Results:** The participant characteristics at baseline were as follows: 53.5 ± 11.6 yrs, 53% f, BMI 32.5 ± 8.4, 42% ex-smokers, 15.1 ± 8.8 months since initial COVID-19 infection, low normal pulmonary function testing, $\dot{V}$O_2peak_ 19.3 ± 5.1 mL/kg/min, 87 ± 17% predicted. After exercise training, participants significantly increased their peak work rate (+16 ± 20 W, *p* = 0.010) and $\dot{V}$O_2peak_ (+1.55 ± 2.4 mL/kg/min, *p* = 0.030). Patients reported improvements in fatigue severity (−11%), depression (−42%), anxiety (−29%), and dyspnea level (−46%). There were no changes in 6MW distance or physical activity. The circulating number of CD3+, CD4+, CD19+, CD14++CD16, and CD16++CD14+ monocytes and CD56+ cells (assessed with flow cytometry) increased with acute exercise (rest to peak) and was not diminished or augmented by exercise training. Plasma concentrations of TNF-α, IL-6, IL-8, IL-10, INF-γ, and INF-λ were normal at study entry and not affected by training. **Conclusions:** Aerobic endurance exercise training in individuals with LC delivered beneficial effects on cardiorespiratory fitness, quality of life, anxiety, depression, and fatigue without detrimental effects on immunologic function.

## 1. Introduction

As the initial waves of acute coronavirus disease 2019 (COVID-19) infection subsided [1], a dark ‘second act’ appeared in 5–30% of COVID-19-infected patients, namely the persistence of viral-type symptoms beyond 12 weeks, even in those with mild disease courses. The most common debilitating ‘long COVID’ (LC) symptoms are fatigue (defined as a sensation of extreme and persistent tiredness or lethargy that hinders normal physical activity) and exercise intolerance (defined as an abnormally low capacity for endurance exercise); however, a myriad of other symptoms related to almost every organ system can occur, including shortness of breath, anxiety, cognitive dysfunction (‘brain fog’), headaches, palpitations, muscle aches, transient rashes, joint pains, sleep disturbances, post-exertional malaise (PEM), clotting abnormalities, chest pains, etc. [2,3,4]. Although the LC etiology is currently unknown, it is thought to be related to one or more pathophysiologic mechanisms involving ongoing viral replication, immune hyperstimulation, dysregulation of the RAAS system, changes in the microbiome, etc. [2]. The trajectory for improvement from LC is highly variable, ranging from complete recovery to prolonged disability with a reduced quality of life and potentially premature death [5]. Currently, there are no specific treatments directed at LC, although a number of studies are underway [6].

Pulmonary rehabilitation (PR) is very well established as being beneficial following high-severity acute COVID-19 (including hospitalization, high-flow oxygen therapy, ICU care, intubation, and mechanical ventilation) [7,8,9]. PR improved anxiety, depression, brain fog, and 6min walk distance (6MWD) in these high-severity groups [8,10,11,12,13,14,15]; however, the limitation in these studies was that 6MWD provides a very limited insight into the mechanism of cardiorespiratory performance or the physiologic mechanisms behind exercise training improvements.

We sought to improve the physiologic and immunologic understanding of outpatient (non-severe) LC. Cardiopulmonary exercise testing (CPET) is an important tool to assess dyspnea, prescribe exercise training, and exclude comorbid diseases causing similar symptoms to LC. CPET is also useful to provide insight into the mechanism(s) of exercise limitation. Prior CPET studies in LC [16,17,18] show baseline reductions in peak pulmonary oxygen uptake ($\dot{V}$O_2peak_) and pulmonary function (both to roughly 80–90% of predicted), even 6–12 months after acute infection.

Recent exercise training and rehabilitation studies in LC utilizing CPET have reported improvements in peak oxygen uptake, work rate, and quality of life [19,20,21,22] in less severe populations (primarily outpatient COVID-19), but have not investigated the inflammatory state or the effects of aerobic exercise training on immune function, as suggested in early 2021 publications by da Silveira and Nieman [23,24].

The evidence exists that endurance and/or strength exercise training may also improve immune function and surveillance (transient increases in the number of NK cells, cytotoxic T cells, and immature B cells), immunoglobulin levels, and reduce inflammatory CRP levels in patients with chronic diseases [24,25]. However, concerns remain as to whether exercise testing and exercise training in LC are safe, because they have the potential to increase post-exertional malaise (PEM) and worsen immune function [26], as can observed in other post-viral syndromes [27].

Our aim in this pilot study was to determine whether moderate aerobic exercise training mitigates LC symptomatology and improves cardiorespiratory fitness and psychological wellness. We hypothesized that LC patients would benefit from a comprehensive moderate aerobic and exercise training program (individualized to baseline CPET) without detrimental effects on immune function.

## 2. Methods and Materials

This study was approved by the Institutional Review Board of The Lundquist Institute for Biomedical Innovation at Harbor–UCLA Medical Center (#32558-01; ClinicalTrials.Gov Identifier: NCT05398692). Written informed consent was provided and documented prior to participation.

### 2.1. Study Design

This was a single-center, pilot clinical trial conducted between February 2022 and December 2023. The study design, interventions, and flow diagram are presented in Figure S1.

### 2.2. Study Enrollment Criteria

The inclusion criteria included adults who were ≥18 years of age, who suffered a COVID-19 infection > 12 weeks prior to study participation (documented by PCR or by patient report when testing was not available), and who experienced one or more of the following persistent symptoms: fatigue, dyspnea, exercise intolerance, PEM, and/or difficulty breathing. Exclusion criteria included subjects unable to perform a technically acceptable PFT or CPET, a desaturation below 80% during exercise, the recent completion of a pulmonary rehabilitation program, a recent or concurrent interventional clinical trial, pregnant or nursing women, malignancy within the past 2 years, injectable insulin use, HIV, system corticosteroids, or any significant respiratory disease other than LC that would put the subject at risk by participating in the study.

### 2.3. Screening Visit

After obtaining informed consent, a detailed history and focused physical examination were performed, including vaccine status, a medical record review, and smoking history. Resting blood pressure (WelchAllyn, SureBP, New York, NY, USA), heart rate, oxygen saturation by portable pulse oximeter (Santa Medical, Tustin, CA, USA), and 12-lead ECG (CardioTech, GT300, Nundah, QLD, Australia) were measured. Peripheral blood laboratories were drawn at the screening visit to ensure the potential subject was safe to enter the study. These included a complete blood count with differential (CBC_diff_), renal, and liver function tests. In addition, D-dimer, Brain Natriuretic Peptide (ProBNP), and inflammatory markers (Ferritin, CRP, and high-sensitivity Troponin (to exclude myocarditis)) were obtained to assess the inflammatory state of the subject. A 10 min NASA lean test [28] was also performed to exclude postural orthostatic hypotension and heart rate autonomic dysfunction.

A pre-bronchodilator spirometry, body plethysmography, and the single breath diffusing capacity of the lung for carbon monoxide (DL_CO_) were measured according to ATS/ERS standards [29,30,31,32]. The post-bronchodilator spirometry was measured 20 min after the inhalation of 4 puffs of albuterol (400 mg). Predicted spirometry values were obtained from NHANES III [33]; lung volumes from ERS [34]; and DL_CO_ from Cotes et al. [35]. The 6MW was assessed per ATS/ERS guidelines [36].

### 2.4. Cardiopulmonary Exercise Testing (CPET)

A ramp incremental CPET at 10–20 watts/min (depending on individual fitness) was performed using an electromagnetically braked cycle ergometer (Excalibur Sport PFM, Lode, Groningen, NL, USA). Intolerance was assessed by the inability to maintain a pedaling cadence > 50 rpm despite encouragement. Gas exchange and ventilatory variables were measured breath by breath (Ultima CPX, MGC Diagnostics, St Paul, MN, USA), while participants breathed through a mouthpiece with a nose clip in place. Heart rate, rhythm disturbances, and ST-T wave changes were monitored using an integrated continuous 12-lead ECG (Mortara, Milwaukee, WI, USA). Exercise blood pressure (Suntech Tango, Morrisville, NC, USA), ratings of perceived dyspnea (RPE_dyspnea_), and leg fatigue (RPE_legs_) (modified Borg CR-10 scale [37]) were assessed every 2 min. The gas exchange lactate threshold (GE-LAT) was assessed using the V-slope method and corroborated with ventilatory equivalents ($\dot{V}$_E_/$\dot{V}$O_2_ and $\dot{V}$_E_/$\dot{V}$CO_2_) and end-tidal partial pressure responses (P_ET_O_2_ and P_ET_CO_2_) [38]. The dead space to tidal volume ratio (V_D_/V_T_) was assessed using transcutaneous PCO_2_ as a valid substitute for arterial PCO_2_ in the mass balance equation (Tosca 500, Radiometer America, Brea, CA, USA) [39,40]. Predicted values for exercise variables were from Hansen et al. [41].

### 2.5. Overnight Oximetry

Overnight oximetry was performed on the night following the screening CPET using a WristOx_2,_ (Model 3150, Nonin Medical, Plymouth, MN, USA) to exclude sleep-disordered breathing.

### 2.6. Patient Reported Outcomes

Participants completed the Short Form Health Survey (SF-36), Fatigue Severity Score (FSS), Patient Health Depression Questionnaire (PHQ-9), General Anxiety Disorder (GAD-7), modified Medical Research Council Dyspnea Scale (mMRC), mini Mental Status Examination (MMSE), Post COVID-19 Functional Status (PCFS), modified DePaul Symptom Questionnaire—Post-Exertional Malaise (mDSQ-PEM), and 7-Day Symptom Diary. See Supplementary Materials for details.

### 2.7. Physical Activity

Seven-day physical activity was assessed using a waist-worn triaxial accelerometer (Dynaport, McRoberts, The Hague, NL, USA). Participants were instructed to wear the activity monitor for 24 h except when bathing or showering. At least 8 h of active time wear, over at least 4 days, was required for measurement validity.

### 2.8. Immunophenotyping and Cytokine Analysis

Venous blood samples were obtained at rest and at peak exercise during the pre-training and post-training CPET. The enumeration and phenotyping of major leukocyte populations present in the circulation—neutrophils (CD16+ cells), monocytes (CD14++16−, CD16++CD14+, CD80+, CD86+, CD80+CD86+), T cells (CD3+ T, CD4+ T, CD8+ T, CD4+FOXP3+ Tregs), B cells (CD19+ B, CD19+CD24+CD38+ Bregs), and NK cells (CD56+ NK, CD56^hi^CD16− NK, CD56^low^CD16+ NK)—were performed based on methods previously described [42] with minor modifications (see Supplementary Materials). Plasma TNF-α, IL-6, IL-10, IL-8, IFN-γ, and IFNL1 concentrations were measured using a commercially available ELISA (see Supplementary Materials).

### 2.9. Exercise Training

Participants performed 10 weeks of twice-weekly, 1.5 h, on-site aerobic training sessions involving cycle and treadmill ergometers. Two types of ergometers were used for subject training (combined equally for each session) to break up the monotony of exercise training and involve different muscle groups. All training sessions were directly supervised. The initial exercise time was 10 min, and participants were verbally assessed for PEM within 48 h of each session, either at their next visit or by phone. If PEM occurred or worsened, the exercise intensity and time were decreased by 50%. If no PEM occurred, the intensity and exercise duration were gradually increased (up to 60 min, treadmill and cycle ergometer combined) by the end of the 10-week training. Cycle ergometer training started at an intensity that was 25% of the initial CPET peak work rate and progressed from a constant work rate to interval training as tolerated. Treadmill constant work rate training was initiated at a 1 MPH speed, 0% incline, and 5 min duration and progressed as individually tolerated. RPE targets were 3–5 during training. A warm up and warm down were performed, and participants were educated with respect to breathing techniques to reduce dyspnea and anxiety, training with Therabands^®^ (Akron, OH, USA), gentle yoga, stretching, balance exercises, and relaxation techniques, as well as given information on nutrition, hydration, and self-care [43].

### 2.10. Post-Training Assessments

The CPET, patient reported outcomes, physical activity, and immunologic assessments were repeated at the end of the 10-week training program.

### 2.11. Statistical Analyses

Data are presented as mean ± SD unless otherwise stated. Statistical significance was accepted at *p* < 0.05. Data were checked for data normality using the Shapiro–Wilk test, histograms, Q-Q plots, and boxplots. As this was a pilot study, no formal a priori sample size calculation was performed. SPSS (version 24.0; IBM, Chicago, IL, USA) was used for statistical analyses.

For the CPET, paired *t*-tests were used to assess pre- and post-training differences in cardiorespiratory and perceptual responses during the CPET. For questionnaires, paired *t*-tests assessed changes in depression and anxiety. Wilcoxon signed rank tests assessed changes in fatigue, dyspnea, the magnitude of post-COVID-19 functional status, and cognitive impairment, as the data were not normally distributed. Changes in SF-36 items were analysed using paired *t*-tests, except for two items that were not normally distributed, where non-parametric sign tests were used. An exact McNemar’s test determined pre- and post-training changes in the proportion of participants reporting PEM. A 2-way repeated-measure ANOVA (day × training) analyzed pre- and post-training differences in 7-day symptom diary items pre- and post-training. When the sphericity assumption was not met, the Greenhouse–Geisser correction was applied.

Two-way repeated-measure ANOVAs (acute exercise × training) were used to assess immune cell number and cytokine concentration pre- and post-training differences immediately before CPET (i.e., rest) and at the peak CPET work rate. When the sphericity assumption was not met, the Greenhouse-Geisser correction was applied. Significant acute exercise and training interaction effects were followed up using paired *t*-tests and Fisher’s LSD.

## 3. Results

### 3.1. Participants

Twenty-one participants consented. The enrollment chart is presented in Figure S2. Five participants were excluded from the study intervention and referred for medical evaluation due to abnormal screening tests, including very high glucose (one); an elevated BNP (one); an ischemic exercise ECG during CPET (one); a high-degree heart block with bradycardia (one); and repeated COVID-19 infection after the first rehabilitation visit (one). Two participants withdrew prior to exercise training: one due to the perceived risk of contracting COVID-19 during exercise training and one due to perceived increased PEM symptoms risk with exercise training. Fourteen participants completed the exercise training study.

### 3.2. Demographics

Demographics, blood chemistry, CBC_Diff_, and PFT results at screening are reported in Table 1 for the 14 participants who completed the study. The screening tests were all within normal limits (Table 1). There were no differences in screening test results between those participants who completed the study (n = 14) and those who screen-failed or dropped out (n = 7) (Table 1). No subject had sleep-disordered breathing suggested from the overnight oximetry.

### 3.3. Exercise Training Compliance

Participants completed 96% of onsite visits during the 10-week, 2 sessions per week, 1.5 h session exercise training program. Twelve sessions (4% of the total visits) were allowed to be completed off-site to accommodate patient travel or weather. Participants started at a low exercise duration and intensity (5–10 min) and advanced, with the goal of reaching 60 min of combined bike and treadmill aerobic exercise by the 10th week. Across all 20 sessions, participants exercised for an average of 43 ± 9 min per session (range 30 to 58 min) and in the final exercise training session, participants averaged 56 ± 7 min of exercise.

### 3.4. CPET Pre- and Post-Training

**Figure 1:**
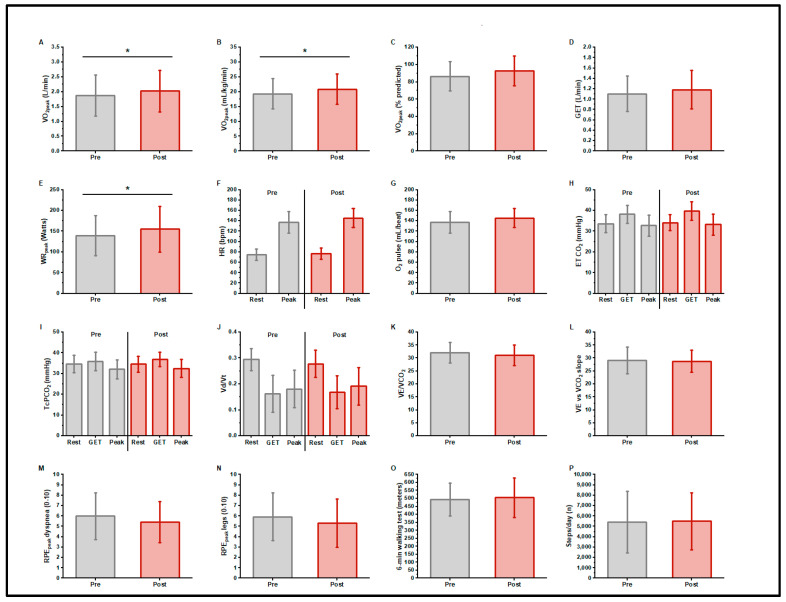
Cardiopulmonary exercise test responses, 6 min walk distance, and daily step counts, before and after exercise training in long COVID patients. n = 14; $\dot{V}$O_2peak_, gas exchange threshold (GET), and peak work rate increased with training (**A**–**E**) (* *p* < 0.05). There was no change in resting or peak heart rate or peak O_2_ pulse with training (**F**,**G**). Indices of ventilatory efficiency did not differ with training (**H**–**L**). There were no differences in peak exercise ratings of dyspnea or leg fatigue with training (**M**,**N**). There was no change in 6 min walk distance (**O**) or daily step count (**P**) with training.

Table 2 and Figure 1 present CPET responses pre- and post-exercise training. Participants began the trial with an impaired cardiorespiratory function, i.e., $\dot{V}$O_2peak_ (1.88 ± 0.69 L/min, 86.5 ± 16.8% predicted [41], 19.3 ± 5.1 mL/kg/min) and GELAT (1.11 ± 0.34 L/min, 58.7% $\dot{V}$O_2peak_); the peak work rate was 139 ± 49 Watts. The mean ventilatory efficiency was normal: the $\dot{V}$_E_/$\dot{V}$CO_2_ at LAT was 31.9 ± 4.0, the $\dot{V}$_E_ to $\dot{V}$CO_2_ slope was 29.0 ± 5.3. The resting transcutaneous PCO_2_ was 34.7 ± 4.2 mmHg. The mean V_D_/V_T_ at GELAT was normal at 0.16 ± 0.07. The peak Borg scores were RPE_dyspnea_ 5.9 ± 2.3 and RPE_legs_ 6.0 ± 2.3. After training, the $\dot{V}$O_2peak_ was 2.03 ± 0.70 L/min, increasing by a mean of 150 ± 200 mL/min (7.9% increase, *p* = 0.017) or 1.51 ± 2.3 mL/kg/min (8% increase, *p* = 0.030). The peak work rate increased 16 ± 20 watts (11.5% increase, *p* = 0.010). There were no significant changes in indices of ventilatory efficiency ($\dot{V}$_E_/$\dot{V}$CO_2_ at LAT, or $\dot{V}$_E_ to $\dot{V}$CO_2_ slope) or V_D_/V_T_. At peak exercise, RPE_dyspnea_ (5.4 ± 2.0), and RPE_legs_ (5.3 ± 2.3) did not differ in response to training.

### 3.5. 6MWD

The mean 6MW distance did not significantly change after exercise training (Table 2, Figure 1O).

### 3.6. 7-Day Activity Monitoring

There was no change in the daily step count after exercise training. Total energy expenditure and time during sedentary, light, moderate, and vigorous activity also did not change significantly (Table 2, Figure 1P).

### 3.7. Patient-Reported Outcomes/Questionnaires

Most SF-36 subscale scores (Table 3) improved (statistically and with respect to minimally important differences) with exercise training. Improvements were observed in physical functioning, energy/fatigue, emotional well-being, social functioning, and general health. Physical and emotional role limitations and pain changes were not changed with exercise training.

Exercise training was associated with a significant reduction in depression scores (PHQ-9) from moderate to mild, while anxiety (GAD-7) was unchanged (mild, Table 3). There was an important MCID reduction in mean FSS, although this was not statistically significant. There was an MCID reduction in the mMRC dyspnea score after exercise training (Table 3). The statistically significant increase in the MMSE score failed to reach the accepted MCID of 1.5 to 2.0 (Table 3). The PCFS decreased significantly from median; however, an MCID is not well established for this questionnaire.

By mDSQ-PEM criteria, 78.6% of our participants (11/14) qualified as having PEM in the past 6 months based upon a ≥2 score (on a 0–4 range) in both frequency and severity in at least one of the five PEM-related questionnaire items. Nine participants reported a PEM score ≥ 2 after training (64.3%), which was not different from the baseline (*p* = 0.500).

In some exercise training sessions, participants self-reported lingering fatigue from the prior training session, consistent with the concept of post-exertional symptom exacerbation (PESE) or PEM. Across the study, this occurred an average of 3 sessions out of 30 (range 0–7). PESE rarely required training intensity reduction, and the symptoms responded by the next training visit.

The 7-day symptom diary tracked daily fatigue, shortness of breath, anxiety, cough, brain fog, overall health, and activity levels (Figure S3). Symptoms of fatigue, brain fog, and overall health were significantly reduced following exercise training, but the remainder of the symptom scores, although lower post-training, did not differ statistically.

## 4. Immunologic Measurements

### 4.1. White Blood Cell (WBC) Numbers

**Figure 2:**
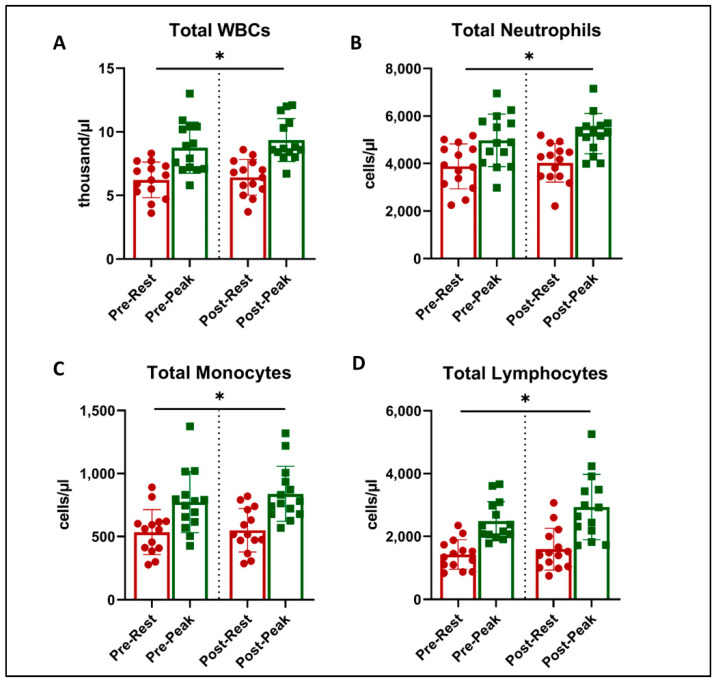
Total leukocyte counts at rest (red) and peak exercise during CPET (green) before and after exercise training in long COVID patients. There was an increase in cell numbers related to acute exercise in total WBC counts (**A**), total Neutrophils (**B**), Total Lymphocytes (**C**), and Total Monocytes (**D**) (* = all *p* < 0.001, 2-way ANOVA). There was no effect on this response with exercise training.

There was a main effect of acute exercise on WBC counts (ANOVA *p* < 0.05), with expected and significant increases from rest to peak exercise during CPET (Figure 2). However, there was no effect of exercise training on WBC counts or an interaction. 

### 4.2. Immune Cell Subsets

**Figure 3:**
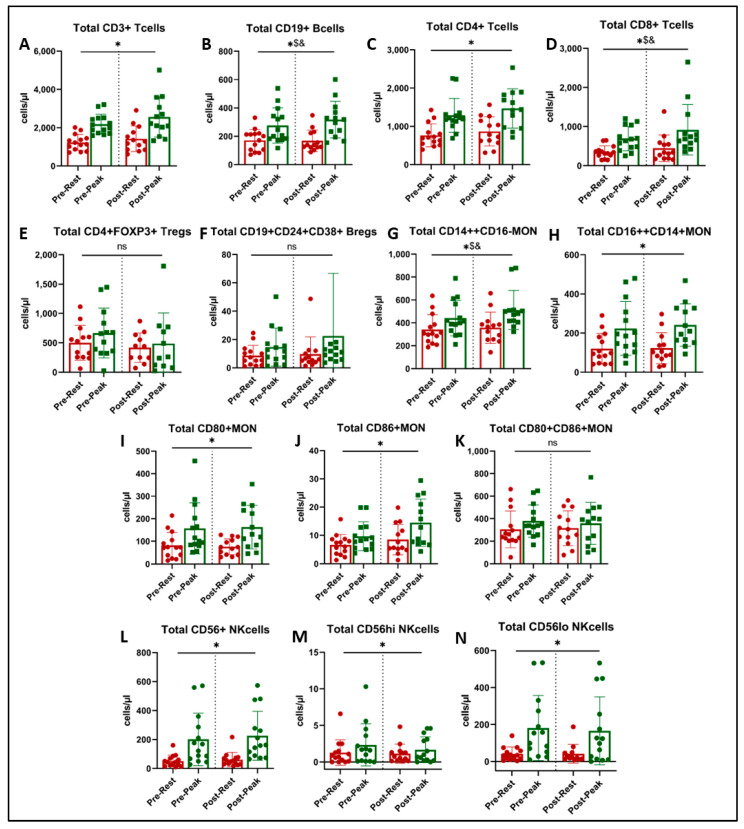
Immune cell subtype counts at rest (red) and at peak exercise during CPET (green) before and after exercise training in long COVID patients. * = main effect of acute exercise (**A**,**C**,**H**,**I**,**J**,**L**,**M**,**N**). $ = acute exercise x training interaction (**B**,**D**,**G**). & = *p* < 0.05 in paired *t*-test (post-hoc, (**B**,**D**,**G**)). There was no effect of exercise training on immune cell subset counts (ns = not significant, panels **E**,**F**,**K**).

There was a significant main effect of acute exercise on CD3+ T cells (*p* < 0.001, Figure 3A), CD4+ helper T cells (*p* < 0.001, Figure 3C), CD16++CD14+ non-classical monocytes (*p* < 0.001, Figure 3H), CD80+ and CD86+ monocytes (*p* < 0.001, Figure 3I,J), and CD56+ NK cells and their two subsets (*p* < 0.001, Figure 3L–N). There was no effect of acute exercise or exercise training on FOXP3+ regulatory T cells (Figure 3E), CD24+CD38+ regulatory B cells (Figure 3F), or CD80+CD86+ activated monocytes (Figure 3K).

We found a significant interaction (acute exercise x training) on the number of CD19+ B cells (Figure 3B, *p* = 0.030) and CD14++CD16− classical monocytes (Figure 3G, *p* = 0.043). Post-hoc analysis showed that the acute exercise response in CD19+ B cells at peak was greater post-training (Figure 3B, *p* = 0.033), and peak CD14++CD16− classical monocytes approached significance (Figure 3G, *p* = 0.051).

### 4.3. Plasma Biomarkers and Cytokines

**Figure 4:**
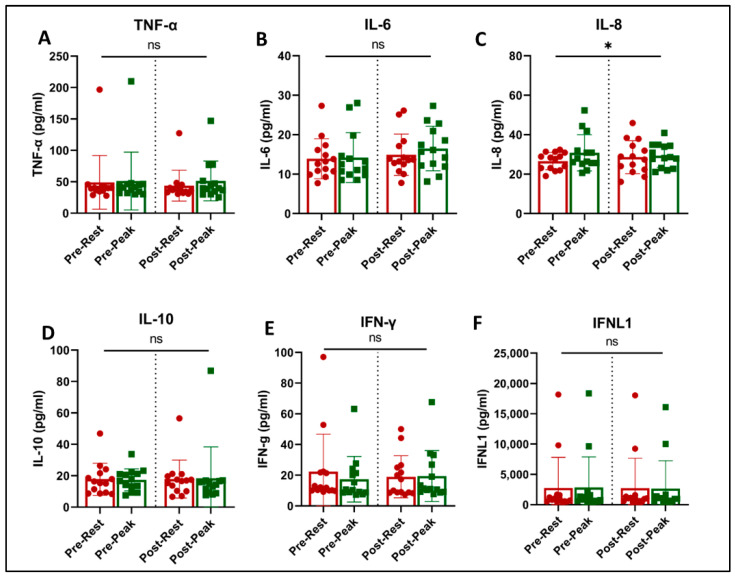
Plasma immune biomarkers at rest (red) and at peak exercise during CPET (green) before and after exercise training in long COVID patients. * = slight increase in IL-8 with acute exercise (*p* = 0.042, (**C**)). There was no effect of training on any of the evaluated immune biomarkers. ns = not significant.

Acute exercise and training effects on plasma inflammatory biomarkers and cytokines are shown in Figure 4. Acute exercise increased the level of IL-8 (*p* = 0.042, Figure 4C) and showed a trend towards an increase in IL-6 concentrations (*p* = 0.061, Figure 4B). There was no effect of acute exercise or exercise training on the levels of TNF-α, IL-10, INF-γ, and INFλ (1FNLl).

## 5. Discussion

Our pilot study investigated the effects of a CPET-based exercise training program on physiologic, immunologic, and patient-reported outcomes in LC. We found that exercise training was well tolerated and resulted in increased cardiorespiratory fitness and marked improvements in symptoms and quality of life without adverse immunologic function effects. The pathophysiologic mechanism(s) of the observed limitations and subsequent improvements with exercise training observed in this study are not well understood. The proposed mechanisms for long COVID include persistent viral replication, ongoing immune overstimulation, dysregulation of the RAAS system, changes in the microbiome, and changes in organ function from COVID-19 infection [44]. Clearly, several of these mechanisms could be affected by exercise training, and future studies will hopefully further elucidate the contributions of these various mechanisms.

Our LC participants had an impaired $\dot{V}$O_2peak_ at baseline, as is observed in the LC CPET literature; however, 20 sessions of CPET-based exercise training over 10 weeks significantly increased the $\dot{V}$O_2peak_ and peak work rate. Training was individualized to the initial CPET results and was begun at low intensity to avoid PESE/PEM. We encountered PESE/PEM on average in 3 of the 20 training sessions per participant, and in these cases, we scaled back the training prior to slowly advancing again. Despite a low initial training duration (10 min) and intensity (25% of the initial CPET peak work rate), with occasional intensity decreases due to symptom exacerbation, participants reached an average training session duration of 43 ± 9 min of aerobic exercise per session over the 20 sessions and 56 ± 7 min at the end of the training program. Overall, a CPET-guided exercise prescription of aerobic exercise training with symptom education was effective in increasing cardiorespiratory fitness in LC patients.

We did not find any substantial ventilatory or gas exchange abnormalities in our subjects. Specifically, we found that the gas exchange efficiency assessed by $\dot{V}$_E_/$\dot{V}$CO_2_, the $\dot{V}$_E_-$\dot{V}$CO_2_ slope, and V_D_/V_T_ were normal prior to training and did not change with training. The P_TC_CO_2_ and P_ET_CO_2_ values were slightly low (~34–35 mmHg) at rest but behaved normally with exercise.

An important point of this pilot study is that CPET prior to an exercise training intervention in those with LC can uncover unexpected comorbidities attributed to LC symptoms (e.g., fatigue, dyspnea, exercise intolerance, PEM, etc.) We excluded participants with potential contraindications to exercise training, including myocarditis (troponin and BNP), autonomic insufficiency (NASA lean test), and serious comorbidities that might masquerade as LC symptoms (e.g., coronary artery disease, cardiac rate disturbances, metabolic disorders) by performing a CPET prior to exercise training. These screening procedures identified 5 of 21 patients (25%) requiring a medical referral and evaluation, with two situations being life threatening. In addition, the similarity between LC symptoms of fatigue, exercise limitation, and chest pains and the symptoms of significant cardiovascular or metabolic disease warrants detailed investigation prior to exercise training and may lead to an alternative diagnosis from LC. We therefore strongly believe that all LC patients should undergo detailed screening procedures including CPET before embarking on new exercise training programs.

Arguments against performing CPET testing or exercise training in LC relate to the findings with other post-viral syndromes (e.g., Myalgic Encephalomyelitis/Chronic Fatigue Syndrome/) where structured exercise may worsen PEM. In this study, we were careful to assess for PEM at entry with a standardized questionnaire (DePaul, retrograde to 6 months) and review any new or worsened symptoms within 48 h of each exercise training session. The recall range of 6 months for the DePaul questionnaire may be judged too wide; however, we wanted to ensure that we had the largest range of understanding of our participant’s symptomatology. Although we found that 11 out of 14 participants identified PEM > 2 using the DePaul questionnaire criteria, being sensitive to symptom fluctuations in our approach, including flexible adjustments of training intensity and duration, allowed participants to increase their cardiorespiratory fitness in response to an aerobic exercise training program without an increase in PEM. A recent review of exercise training (with and without PEM) in LC by Gloeckl, et al. provides evidence-based practical advice regarding the optimal exercise program, including aerobic training, resistance exercise, and inspiratory muscle training programs for long COVID, with and without PEM, similar to the current study [43].

Patient-reported outcomes showed marked quality of life improvements, including improvements in physical functioning, energy/fatigue, emotional well-being, social functioning, and general health (SF-36) and reductions in depression (PHQ-9). In addition, functional disability due to dyspnea (mMRC), and cognitive impairment (MMSE) improved (Table 3).

We hypothesized that the immunophenotype and balance of inflammation/anti-inflammatory forces in LC would improve with aerobic exercise training. This was based on prior studies showing that exercise training enhances the innate immune function and reduces systemic inflammation in other chronic metabolic and inflammatory diseases [45,46,47]. However, we did not observe significant changes in circulating cell counts or immune biomarkers with exercise training. Nevertheless, there was a transient increase in immune cell subtypes involved in exercise-induced immunosurveillance [45,48]. This aligns with previous research indicating that exercise boosts the recirculation of cytotoxic T cells, immature B cells, and NK cells, which can augment immune surveillance and the suppression of tumorigenesis [46], and similar immune improvements have been demonstrated in other conditions [49,50]. Thus, each bout of moderate exercise appears to temporarily enhance immunosurveillance, whereby regular exercise may provide multiple health benefits, including reduced morbidity and lower systemic inflammation [24].

Our results suggest that the transient change in immune cell subpopulations and biomarkers due to acute exercise in our LC participants was not adversely affected by exercise training and resulted in significant cardiorespiratory fitness and quality of life improvements. In fact, fatigue at entry and fatigue improvements with training did not appear to be associated with immune hyperstimulation, suggesting other etiologies of exercise limitation.

## 6. Strengths and Limitations

The strengths of this study include in-depth physiologic and questionnaire screening, CPET testing before and after exercise training, activity monitoring, multiple patient-reported outcome assessments, detailed acute exercise and training immunologic data, and screening for comorbid diseases mimicking LC symptoms. The potential limitations are those related to a pilot study: a small sample size, (exacerbated by screen failures due to previously unidentified medical issues and concerns about reinfection or PEM), no control group (subjects were their own control pre/post training), and potential variability in the participant phenotype, since no gold standard biomarker currently exists for LC syndrome.

## 7. Conclusions

Our hypothesis that LC patients would benefit from a comprehensive moderate aerobic endurance exercise training program prescribed using a baseline CPET was affirmed. Exercise training appears safe to perform in LC patients, when there is great attention to screening for unrecognized medical conditions that may mimic or exacerbate LC symptoms and when the training intensity and duration are flexibly adjusted during the training sessions to limit flare ups of PESE/PEM symptoms. Exercise training substantially improved patient-reported outcomes of mental health, well-being, and symptoms of depression. The resting immune system profile, and the immune response to acute exercise, was unaffected by exercise training despite reductions in fatigue-related symptoms, suggesting that immune hyperstimulation may not be the primary mechanism of LC fatigue symptoms in our study. LC patients benefit from a comprehensive moderate aerobic exercise training program structured to their individual cardiorespiratory fitness, as guided by CPET.

## Figures and Tables

**Table 1:** Baseline demographics, long COVID symptoms, screening laboratories, and pulmonary function testing. n = 14 completed study vs. 7 who did not complete study. All values, mean ± SD. FEV1, forced expiratory volume in 1 s; FVC, forced vital capacity; TLC, total lung capacity; RV, residual volume; DLCO, diffusing capacity of lung for carbon monoxide. * = *p* < 0.05 (n = 7) relative to group who completed study (n = 14).

Demographics	n = 14 Finished Training		n = 7 (Did Not Continue Study)	
	Mean	S.D.	Mean	S.D.
Age (years) ± SD	53.5	11.6	60.3	6.2
% Female (n, %)	6/43		5/71 *	
Race (Asian/Black/Caucasian) %	7/0/93		0/0/100	
Ethnicity (Non-Hispanic/Hispanic) %	57/43		71/29	
Height (cm)	174	10.6	167	15.5
Weight (kg)	98	26.4	83	28.0
BMI (kg/m^2^)	32.5	8.4	29.5	8.4
Smoking Status (Never/Ex) %	58/42		86/14 *	
Time Since Initial COVID Infection (Months)	15.1	8.8	21.7	12.0
Severity of COVID Infection (Non Hospitalized, n, %)	11/79		7/100 *	
Systolic BP (mmHg)	129	14	128	18
Diastolic MB (mmHg)	73	10	79	9
Activity Level at Study Entry (Sedentary/Walking/Regular Workouts) %	29/57/14		43/43/14	
**Primary Long COVID Symptoms (Inclusion Criteria)**	**n**	**%**	**n**	**%**
Fatigue	12	86	7 *	100
Dyspnea	5	36	1	14
Exercise Intolerance	6	46	4	57
Post-Exertional Malaise	8	57	4	57
Difficulty Breathing	5	36	1	14
Brain Fog	14	100	7	100
**Screening Laboratories (Normal Range)**	**Male/Female Mean**	**Male/Female S.D.**	**Male/Female Mean**	**Male/Female S.D.**
Hemoglobin (Range: male 13.2–17.1, female 117–15.5 gm/dL)	14.9/13.4	1.1/0.9	16.3/14.0	0.6/1.0
Hematocrit (male 38.5–50%, female 35–45%)	44.1/39.3	3.0/2.6	47.7/41.8	1.8/2.6
AST (10–30 U/L)	20	6.4	30	29.3
ALT (6–29 U/L)	24	15.2	47	59.9
Bilirubin (0.2–1.2 mg/dL)	0.6	0.3	0.7	0.4
Creatinine (Range: F 0.5–1.1 mg/dL. M 0.7–1.3 mg/dL)	0.9	0.2	0.8	0.2
D-Dimer (normal < 0.5 mcg/mL)	0.44	0.18	1.15 *	0.96
Ferritin (38–280 ng/mL)	105	95	74	48
CRP (normal < 8.0 ng/mL)	4.3	5.8	8.1	9.5
Pro-BNP (normal < 253 pg/mL)	43	49	132	181
High-Sensitivty Troponin (normal < 15 ng/L)	6.8	2.2	6.6	1.8
**Pulmonary Function Testing**	**Mean**	**S.D.**	**Mean**	**S.D.**
FEV_1_ (L)	3.11	0.69	2.61	0.99
FEV_1_ (% predicted)	94	19	91	14
FEV_1_ (% change with BD)	5	5	7	6
FVC (L)	3.89	0.85	3.41	1.36
FVC (% predicted)	92	16	92	10
FEV_1_/FVC	80	8	77	9
TLC (L)	5.42	1.11	5.43	1.76
TLC (% predicted)	86	12	94	9
D_L_CO (mL∙min^−1^∙mmHg^−1^)	23.7	5.9	22.0	7.2
D_L_CO (% predicted)	93	18	96	10
6-Minute Walk Distance (m)	498	105	436	140

* = *p* < 0.05 (n = 7) relative to the group who completed the study (n = 14).

**Table 2:** Pre- and post-exercise training ramp CPET results (n = 14). $\dot{V}$O_2_, oxygen uptake; LAT, estimated lactic acidosis threshold; for $\dot{V}$O_2_ at LAT, the % predicted is 40% of peak $\dot{V}$O_2_; V_E_, minute ventilation; $\dot{V}$CO_2_, carbon dioxide output; $\dot{V}$_E_ to $\dot{V}$CO_2_ slope, the slope of the relationship between $\dot{V}$_E_ and $\dot{V}$CO_2_ measured between 20 W and the respiratory compensation point; WR, work rate (watts); MVV, maximum voluntary ventilation estimated from the forced expiratory volume in 1 s * 40; peak HR, maximum HR recorded during the CPET. Peak O_2_ pulse = peak $\dot{V}$O_2_/peak HR. All predicted values from Wasserman et al. [41].

Exercise and Activity Responses to Training (N = 14)							
Value	Pre	S.D.	Post	S.D.	Absolute Change	% Change	*p* Value
**CPET**							
Peak Oxygen Uptake							
Absolute (L/min)	1.88	0.69	2.03	0.70	0.15 ± 0.20	7.4%	**0.017**
mL/Kg/min	19.3	5.1	20.9	5.1	1.55 ± 2.4	7.4%	**0.030**
% Predicted	87	17	93	17	6 ± 1.0	6.7	0.815
Gas Exchange LAT (GELAT, L/min)	1.11	0.34	1.18	0.37	0.06 ± 0.19	5.1%	0.250
AT as a % of Peak $\dot{V}$O_2_	59		58				
Peak Work Rate (W)	139	49	155	55	16 ± 20	10.3%	**0.010**
Resting Heart Rate (b/min)	75	11	77	11	2 ± 8	2.6%	0.421
Peak Heart Rate (b/min)	137	21	146	18	9 ± 15	6.5%	0.052
% Predicted Peak Heart Rate	83%	14%	88%	10%			
Peak O_2_ Pulse (mL/beat)	13.9	5.2	14.1	5.1	0.11 ± 2.1	0.7%	0.844
Rest ET CO_2_ (mmHg)	33.6	4.3	34.2	3.9	0.6 ± 2.6	1.8%	0.445
GE-LAT ET CO_2_ (mmHg)	38.2	4.4	39.7	4.5	1.5 ± 2.0	3.9%	**0.017**
Peak ETCO_2_ (mmHg)	32.7	5.0	33.2	5.0	0.5 ± 2.3	1.5%	0.457
Rest TcPCO_2_ (mmHg)	34.7	4.2	34.6	3.8	0.08 ± 2.28	0.2%	0.899
GE-LAT TcPCO_2_	35.8	4.4	36.8	3.4	1.0 ± 2.21	2.7%	0.120
Peak TcPCO_2_ (mmHg)	32.1	4.6	32.6	4.4	0.4 ± 2.05	1.2%	0.464
Vd/Vt at Rest	0.29	0.04	0.28	0.05	0.016 ± 0.05	5.5%	0.249
Vd/Vt at GE-LAT	0.16	0.07	0.17	0.06	0.006 ± 0.043	3.7%	0.628
Vd/Vt at Peak	0.18	0.07	0.19	0.07	0.01 ± 0.05	5.5%	0.437
$\dot{V}$_E_/$\dot{V}$CO_2_ @ LAT	32.0	3.9	31.1	3.9	0.9 ± 2.4	2.8%	0.176
$\dot{V}$_E_ to $\dot{V}$CO_2_ slope	29.1	5.1	28.7	4.2	0.37 ± 3.35	1.2%	0.685
Peak RPE1_dyspnea_	6.0	2.3	5.4	2.0	0.6 ± 1.9	10.1%	0.293
Peak RPE2_leg_	5.9	2.3	5.3	2.3	0.6 ± 2.3	10.0%	0.342
**6MW Distance**	498	105	505	123	8 ± 57	1%	0.619
**Activity Monitoring (7 days)**							
Steps/Day (n)	5425	2960	5505	2756	79 ± 1829	1.5%	0.437
Total Estimated Energy Expenditure (Kcal/24 h)	2758	684	2772	601	14 ± 243	−1.2%	0.416
Sedentary Time (hours/minutes)	18:16	3:06	18:29	3:01	−0:04	−0.2%	0.357
Light Activity (hours/minutes)	1:15	0:30	1:15	0:33	0:00	0.6%	0.173
Moderate Activity (hours/minutes)	1:09	0:35	1:03	0:22	−0:03	−0.2%	0.241
Vigorous Activity (hours/minutes)	0:10	0:12	0:25	0:44	0:18	164.4%	0.113

**Table 3:** Patent-reported outcomes: pre- and post-exercise training intervention (n = 14). Patient-reported outcomes: 36-item Short Form Survey (SF-36, higher is better), Fatigue Severity Score (lower is better), Patient Health Questionaire-9 (PHQ-9; for depression, lower is better), General Anxiety Disorder-7 (GAD-7; for anxiety, lower is better), mMRC (lower is better), MMSE (mental status, higher is better), Post-COVID-19 Functional Status (PCFS, lower is better). SF-36 change category, (S = Small, M = Moderate, L = Large).

Patient-Reported Outcomes									
	Pre		Post		Absolute Change	Percent Change	Change Category	MCID	*p* Value
	Mean	S.D.	Mean	S.D.					
**SF-36 (Higher is Better)**									
Physical Functioning	40	21	64	22	24 ± 6.5	60%	M	S 10, M 20, L 30	**0.003**
Energy/Fatigue	24	17	39	23	15 ± 15	63%	S	S 12.5, M 25, L 37.5	**0.002**
Emotional Well-Being	53	25	71	18	18 ± 13	34%	M	S 8.3, M 16.7, L 25	**<0.001**
Social Functioning	38	30	58	28	20 ± 12	52%	S	S 12.5, M 25, L 37.5	**<0.001**
Pain	53	27	63	22	10 ± 18	18%	S	S 10, M 20, L 27.5	0.072
General Health	38	18	51	23	14 ± 18	37%	S	S 10, M 20, L 30	**0.010**
	Median	IQR	Median	IQR			Change Category	MCID (S = Small, M = Moderate, L-Large)	*p* Value
Role Limitations—Physical	0	0	25	75			M	S 12.5, M 25, L 37.5	**0.070**
Role Limitations—Emotional	0	100	33	100			S	S 8.3, M 16.7, L 25	0.125
**PHQ-9 (Depression, Lower is Better)**	12	7	7	6	−5 ± 4	−42%	Moderate- > Mild	5 = Mild, 10 = Moderate, 15 = Mod. Severe, and 20 = Severe)	**0.001**
**GAD-7 (Anxiety, Lower is Better)**	7	6	5	5	−2 ± 4.7	−29%	Mild- > Mild	0–4, Minimal, 5–10 Mild, 10–14 Moderate, and 15–21 Severe Anxiety	0.125
	Median	IQR	Median	IQR			Change Category	MCID	*p* Value
**Fatigue Severity Score (Lower is Better)**	53.5	14.5	44.5	32.2			At MCID	5–11	0.177
**mMRC (Lower is Better)**	2	1	0.5	2			Below MCID	1 unit	**0.015**
**MMSE (Higher is Better)**	28	2	29	2			Below MCID	1.5 to 2.0	**0.010**
**Post-COVID-19 Functional Status (Lower is Better)**	2.5	1	2	2.25			-	Not established	**0.020**

## Data Availability

Data are available with appropriate requests to the corresponding author.

## Supplementary Materials

The following supporting information can be downloaded at: https://www.mdpi.com/article/10.3390/jcm13185590/s1, Figure S1: Study Interventions and Participant Flow by Visit.; Figure S2: Enrollment Flow Chart. Figure S3: Seven Day Symptom Diary; Figure S4: Gating strategy for the lineage panel; Figure S5: Gating strategy for T cells subsets; Figure S6: Gating strategy for regulatory T cells; Figure S7: Gating strategy for regulatory B cells; Figure S8: Gating strategy for the activated monocytes; Table S1: Antibody characteristics used for immune cell phenotyping.
