# Supplementary material for: A Pilot Study on the Effects of Exercise Training on Cardiorespiratory Performance, Quality of Life, and Immunologic Variables in Long COVID
Source: J Clin Med. 2024 Sep 20;13(18):5590. doi: 10.3390/jcm13185590 (PMC11433403; doi:10.3390/jcm13185590)
Supplement: Supplementary file 1 [file jcm-13-05590-s001.zip › jcm-3186872-supplementary.pdf]

Supplement - [JCM] Manuscript ID: jcm-3186872

Title: A Pilot Study on the Effects of Exercise Training on Cardiorespiratory Performance, Quality of Life, and Immunologic Variables in Long COVID

S1: Patient Reported Outcomes

SF-36

The Short Form Health Survey (SF-36) [1] evaluates health status in 7 categories, each from 0% (worst) to 100% (best). The categories and minimum clinically important difference (MCID, S=Small, M=Medium, L=Large) included Physical functioning (S 10, M 20, L 30), Role limitations due to physical health (S 12.5, M 25, L 37.5), Role limitations due to emotional problems (S 8.3, M 16.7, L 25), Energy/fatigue (S 12.5, M 25, L 37.5), Emotional well-being (S 8.3, M16.7, L 25), Social functioning (S12.5, M 25, L 37.5), Pain (S 10, M 20, L 27.5), and General Health (S10, M 20, L 30)[2-5].

Fatigue Severity Score (FSS)

The Fatigue Severity Score [6] has 9 items that are scored on a Likert scale. The minimum score is 9 (least) and the maximum score is 63 (most) and that score is then divided by 9, for a range of 1 to 7. A lower value is better. The MCID is felt to be between 0.5 and 1.2, with 0.08 to 0.4 for improvement, and 1-1.2 for deterioration [7].

Patient Health Depression Questionnaire (PHQ-9)

The Patient Health Depression Questionnaire (PHQ-9) scores nine questions from 0 to 3, with a total score range from 0 to 27[8]. Greater scores are associated with increased risk of depression. Scores of 5, 10, 15 and 20 correspond to mild, moderate, moderately severe, and severe depression. A score of ≥ 10 is associated with a sensitivity of 88% and a specificity of 88% for major depression. An MCID is suggested to be a change of 5 points[9].

General Anxiety Disorder (GAD-7)

The General Anxiety Disorder (GAD-7)[10] scores 7 categories from 0-3, for a total range of 0-21. A score from 0-4 indicates minimal anxiety symptoms, 5-10 mild, 10-14 moderate, and 15-21 severe symptoms. A threshold score of 10 has an 89% sensitivity and 82% specificity for generalized anxiety disorder. The GAD-7 MCID[11] is felt to be a 4 point change.

Modified Medical Research Council Dyspnea scale (mMRC)

A modified Medical Research Counsil (mMRC) Dyspnea scale[12] evaluates dyspnea in patients with respiratory conditions. The scale is from grade 0 ("I only get breathless with strenuous exercise") to grade 4 ("I am too breadless to leave the house, or I am breathless when dressing."). The MCID for the mMRC is one unit change[13,14].

Mini Mental Status Examination (MMSE)

The mini mental status examination (MMSE)[15] consists of 11 questions covering several cognitive domains. The maximum score is 30, and scores above 25 are considered to reflect normal cognitive function[16]. An MCID is considered to be between 1.5 to 2.0[17].

Post COVID-19 Functional Status (PCFS)

A post COVID-19 Functional Status (PCFS) questionnaire[18] is scored between 0 ('I have no limitations in my everyday life and no symptoms, pain, depression or anxiety related to the infection') to 4 ('I suffer from severe limitations in my everyday life: I am not able to take care of myself and therefore I am dependent on nursing care and/or assistance from another person due to symptoms, pain, depression, or anxiety). The MCID has not been established.

Pittsburgh Sleep Quality Index (PSQI)

The Pittsburgh Sleep Quality Index [19] has 19 items that generate seven component scores, subjective sleep quality, sleep latency, sleep duration, habitual sleep efficiency, sleep disturbances, use of sleeping medications, and daytime dysfunction[19]. The sum of the seven components yields one

global score (0 best, 21 worst). 'Poor' sleepers (versus 'good' sleepers) are distinguished by a global score greater than five. The MCID was found to be a change of 4.4 global points in a post-surgical population[20].

DePaul Symptom Questionnaire – Post-Exertional Malaise (DSQ-PEM)

The DePaul Symptom Questionnaire – Post Exertional Malaise (DSQ-PEM)[21] has five questions:

1) Dead, heavy feeling after starting to exercise
2) Next day soreness or fatigue after non-strenuous, everyday activities
3) Mentally tired after the slightest effort
4) Minimum exercise makes you physically tired, and
5) Physically drained or sick after mild activity.

Each question is rated between 0 (none of the time) and 4 (all of the time). The questions have both frequency and severity domains. A threshold of 2 in both frequency and severity for any question indicates the presence of post exertional malaise. Greater summed scores for frequency (0-20) and severity (0-20 range) indicate that more PEM is present. An MCID has not been well established from the ME/CFS literature. The retrograde recall for PEM was 6 months in our survey.

Seven Day Symptom Diary

A Seven Day Diary (example below) was given to each participant concurrent with the 7-day physical activity monitoring. A Likert scale from 0-10 was used for six symptoms, Shortness of Breath, Fatigue, Anxiety, Cough, Brain Fog, and Activity Level. 0 was 'None', 1-2 was 'Very Mild', 3-4 was 'Mild', 5-6 was 'Moderate', 7-8 was 'Severe', and 9-10 'Extremely Severe'.

Visit Date: x/x/x
Subject Initials XXX
Unique Identifier Created XXX-XX

DATE:							
Symptoms Over The Past 24 hours: Rate the severity of your symptoms by filling in a number for each symptom based on the scale below. Make a general decision about how severe each symptom was (as an average) in the last 24 hours.<br><br>**SYMPTOM SEVERITY: 0-None, 1-2Very Mild, 3-4-Mild, 5-6-Moderate, 7-8-Severe, 9-10-Extremely Severe**							
Shortness of Breath							
Fatigue							
Anxiety							
Cough							
Brain Fog							
Activity Level (Low Number – Low Activity, High Number High)							
Overall Health (Low Number - Worse, High Number Better)							

S2: Immunophenotyping and Cytokine Analysis

Reagents for Immunological Measurements

For flow cytometry analyses, the cells were stained using commercially available monoclonal antibodies (Table S1), as previously described [22]. BD FACS lysing solution (BD Biosciences, San Diego, CA, ref. 349202) was used for lysing red blood cells. Fc-receptor blocking antibodies were used (eBioscience ref. 14-9161). Dead cells were excluded using the Fixable Viability Dye (FVD) eFluor 506 (eBioscience, ref. 65-0866) in all panels.

ELISA kits for the detection of human TNF-α (R&D Systems, Inc, Minneapolis, MN, USA, Cat. #PDTA00D), IL-6 (R&D Systems, Cat. #PD6050), IL-10 (R&D Systems, Cat. #PD1000B), IL-8 (R&D Systems, Cat. #PD8000C), and IFN-γ (R&D Systems, Cat. #PDIF50C) were obtained from Bio-Techne (USA). Human IFNL1/Interferon Lambda-1 ELISA kit was purchased from Sigma Aldrich (Saint Louis, MO, USA, lot#0831I0292).

Blood Collection

Blood was collected from the subjects at visits 2 (pre-training) and 24 (post-training). Briefly, a venous cannula was placed into a peripheral vein by an experienced phlebotomist for collection of blood samples before and at peak exercise. At each sample collection, 6mL of blood was collected in a K2-EDTA tube to analyze complete blood count (CBC) with differential and immune cell phenotyping, and 9mL of blood in a K3-EDTA tube was obtained for analysis of cytokines/chemokines. Plasma was separated by centrifugation at 1000 x g for 15 minutes withing 30 minutes of collection. Aliquots of plasma were stored at − 80 °C until analyzed. Blood for immunophenotyping was maintained at room temperature until processing.

Measurement of Plasma Cytokine Concentration

Commercially available high-sensitivity enzyme-linked immunosorbent assay (ELISA) was used to detect and quantify (in duplicate) concentrations of plasma TNF-α, IL-6, IL-10, IL-8, IFN-γ, and

IFNL1/Interferon Lambda-1. The results were measured with the automatic plate reader and calculated based on the measurements of the light absorbance of the tested material.

Flow Cytometric Analysis of Immune Cell Subtypes

K3-EDTA blood was stained with different antibodies to evaluate distinct immune cell subtypes frequency using flow cytometry. Briefly, whole blood (2 mL) was washed (x2) by mixing fresh whole blood and PBS at a 1:1 ratio, followed by centrifugation at 500g for 5 min at 18–22 °C (room temperature). The supernatant was aspirated and discarded, followed by the addition of fresh PBS taking it to the same final volume as input whole blood. Antibodies were shortly spun (about 20 s) and added (1µl) to 200µl of blood aliquoted into Eppendorf tubes. The samples were shortly vortexed and incubated 20 min in the dark at room temperature (RT). 1µl of 1x Fixable Viability Dye solution was added to each sample, followed by incubation for 30 min in the dark at 4 °C. Thereafter, 1 ml of cold PBS (4 °C) was added to the tubes, which were centrifuged for 5 min at 500 x g and the supernatant was aspirated. All samples, irrespective of the panel used, were resuspended in 4000µl of 1x RBC lysing solution, shortly vortexed and incubated 25 min at RT protected from light. After centrifugation for 5 min at 500 x g, the supernatant was aspirated, the samples were resuspended in 300µl staining medium and immediately acquired on the cytometer.

Flow Cytometry Panel Design

To enable detection, enumeration and phenotyping of major leukocyte populations present in circulation — neutrophils, monocytes, T cell, B cells, NK cells — we used a previously published article [22] with minor modification to design four 5-color cytometry panels. The "lineage" panel (Panel A) covered the major cell populations, providing a reference for comparison with other consortia and served as an internal control for other panels (Fig. S4). This Panel characterizes CD3+ T cells, CD19+ B cells,

CD56+ NK cells, 2 subsets of NK cells including CD56hi and CD56low NK cells, CD16+ neutrophils, CD14++CD16- classical monocytes and CD16++CD14+ non-classical monocytes. The "T cell" panel (Panel B) was designed to classify CD4+ helper and CD8+ cytotoxic subsets (Fig. S5). The "Regulatory T cell" panel (Panel C) was designed to characterize CD4+FOXP3+ T cell subsets (Fig. S6). The "B cell" panel (Panel D) identifies CD19+ B cell population and an important subset of CD24+CD38+ Regulatory B cells (Fig. S7). And the Panel E represents the frequency of activated monocytes (CD80+, CD56+, and CD80+CD86+ cells)(Fig. S8).

Gating Strategy

Figure S4 shows the gating strategy for the lineage panel (Panel A) of a representative subject. The gating strategy of other panels (Panel B, C, D, E) have been represented in supplementary document as Figures S5, S6, S7, S8.

Supplementary Figures

Figure S1. Study Interventions and Participant Flow by Visit.

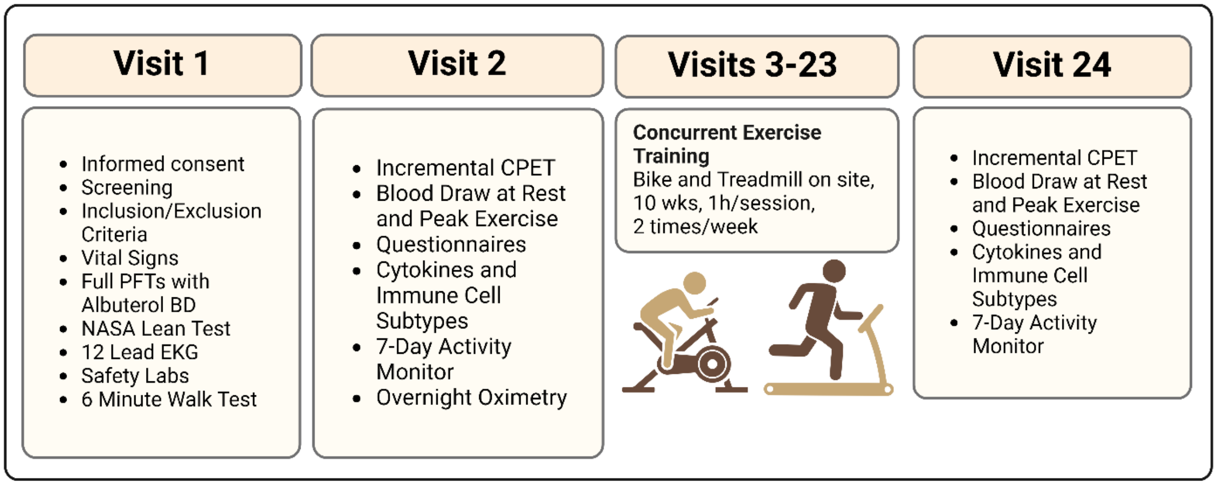

Figure S2. Enrollment Flow Chart.

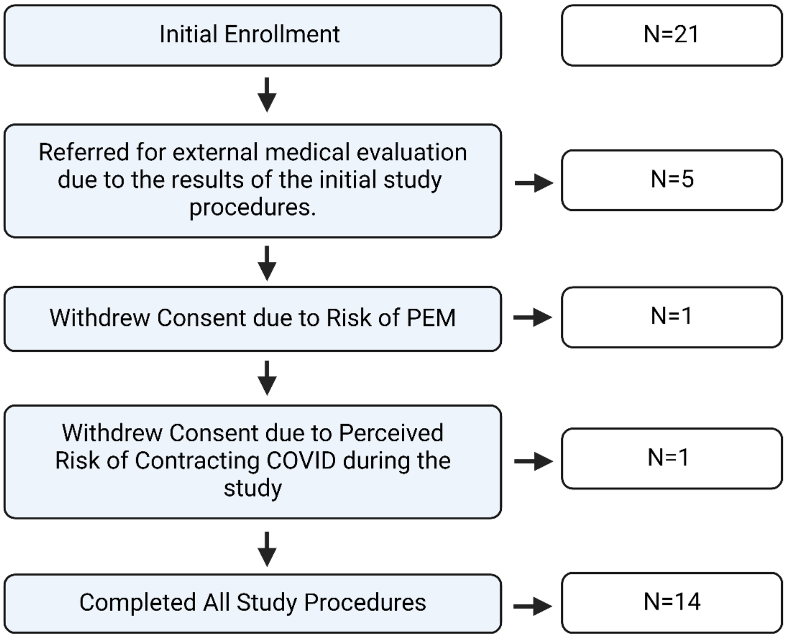

Initial Enrollment
N=21
Referred for external medical evaluation due to the results of the initial study procedures.
N=5
Withdrew Consent due to Risk of PEM
N=1
Withdrew Consent due to Perceived Risk of Contracting COVID during the study
N=1
Completed All Study Procedures
N=14

Figure S3. Seven-day symptom diary. 7-day diary pre and post exercise training assessment of self-reported fatigue, anxiety, shortness of breath, cough, brain fog, overall health, and activity level on a Likert scale of 1-10. Only Fatigue and Brain Fog differed from pre to post exercise training. * = P<0.05

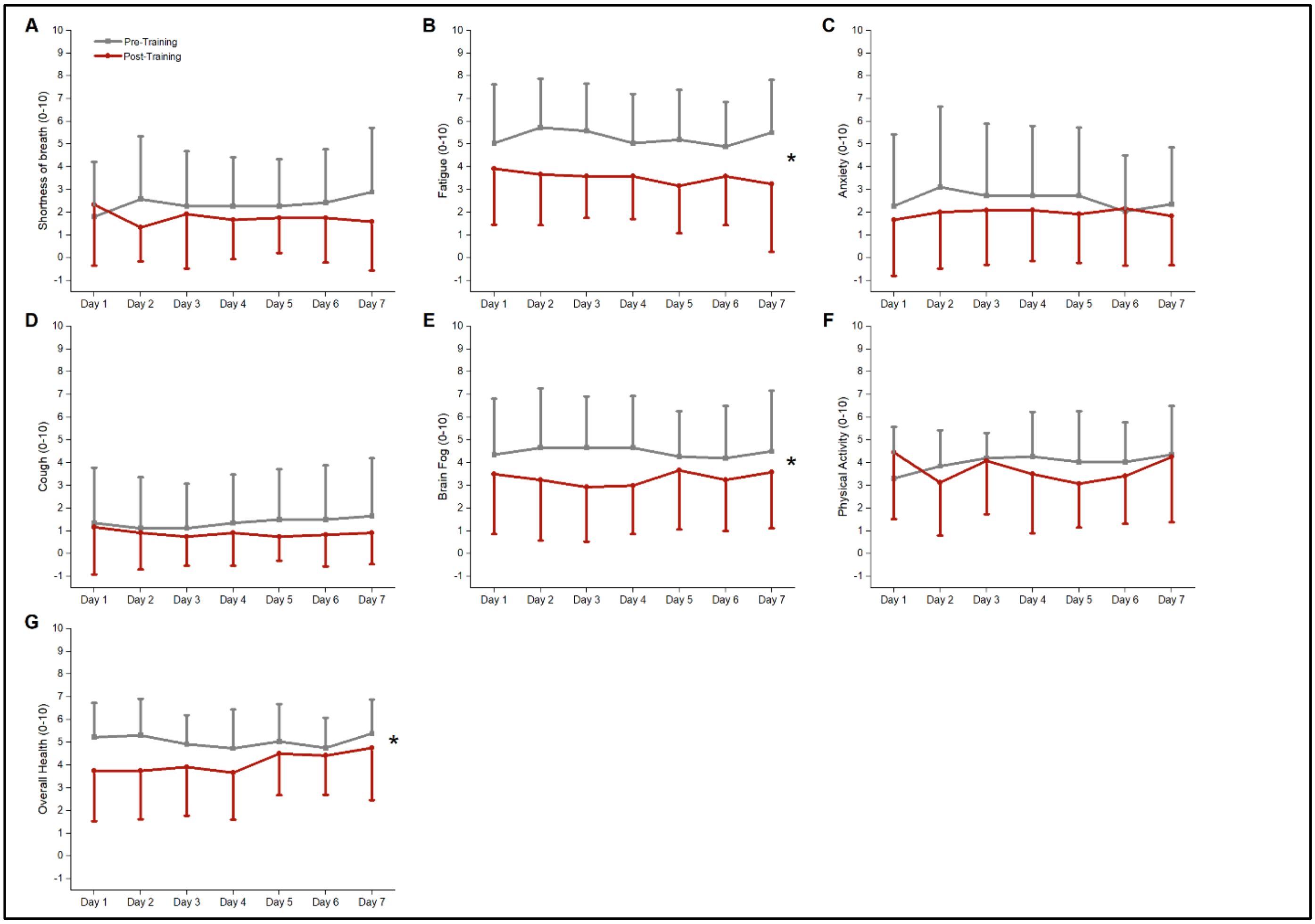

Figure S4. Gating strategy for the lineage panel (Panel A). (**A**) Doublets were excluded from the analysis using forward scatter width (FSC-W) and forward scatter area (FSC-A). This is followed by side scatter height (SSC-H) and side scatter area (SSC-A), then followed by live cells. (**B**) B cells were identified by expression of CD19+ cells (Red gate). T cells were identified within the CD19– cells based on CD3 expression (green gate) and analyzed for the expression of CD4 and CD8

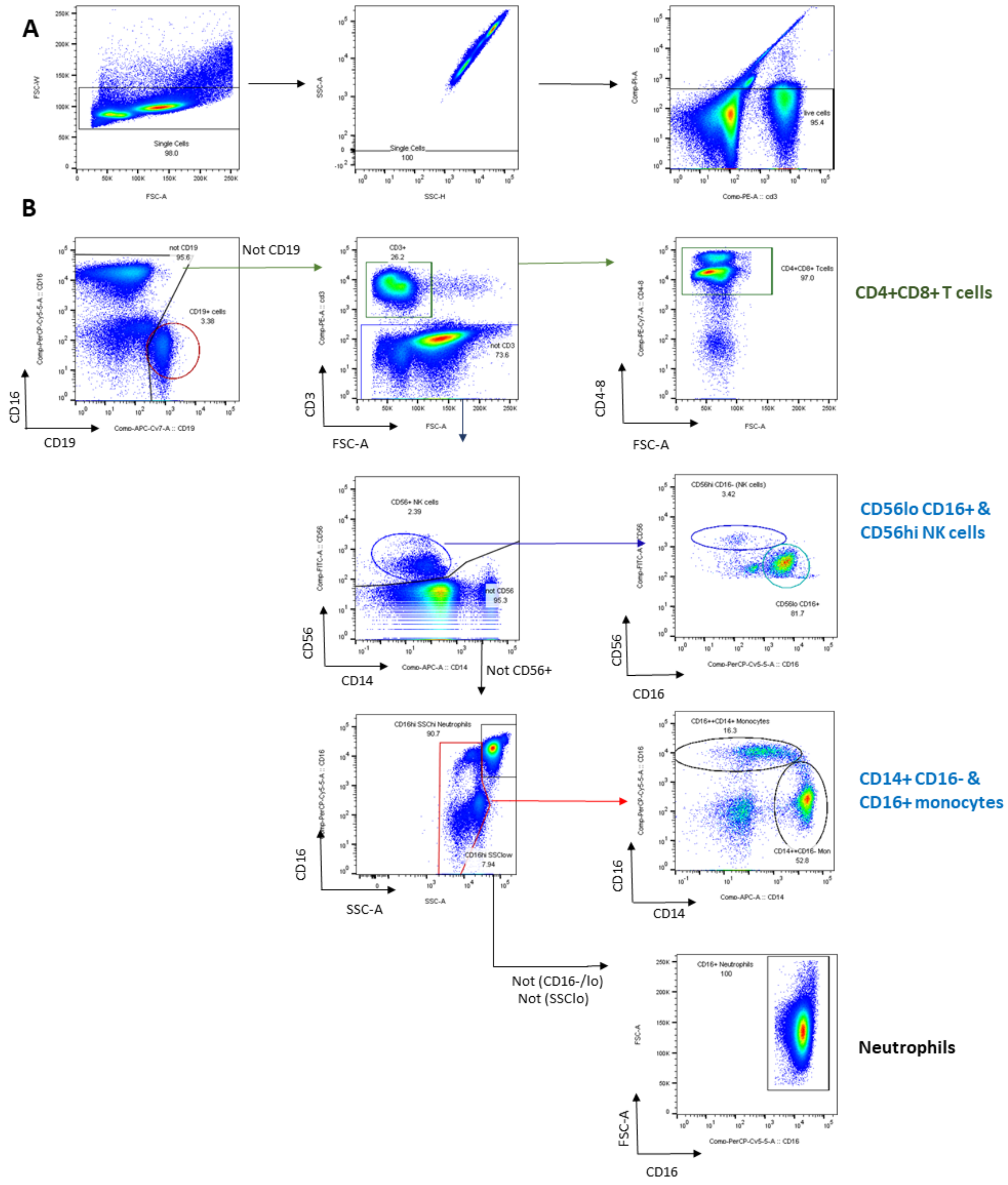

cells. Within the CD3- cells (Blue gate) NK cells were identified as CD56+ and analyzed for their expression of CD16 and CD56. In the population of CD56– cells, CD14+ and CD16+ monocytes were gated within the CD16$^{+/low}$ SSC^{low} cells (Red gates). Finally, neutrophils were identified as CD16+SSChi (Black gate).

Figure S5. Gating strategy for T cells subsets (Panel B). (**A**) Doublets were excluded from the analysis using forward scatter width (FSC-W) and forward scatter area (FSC-A). Single cells were identified by side scatter height (SSC-H) and side scatter area (SSC-A), and then live cells were identified. (**B**) Total lymphocytes were identified for the expression of CD3+ and CD19+ (violet gate). Within this gate (violet gate), the expression of CD3+ T cells (brown gate) and CD19+ B cells (Blue gate). Withing the CD3+ cells, the expression of CD4+ helper T cells (Green gate) and CD8+ cytotoxic T cells (Red gate) were identified.

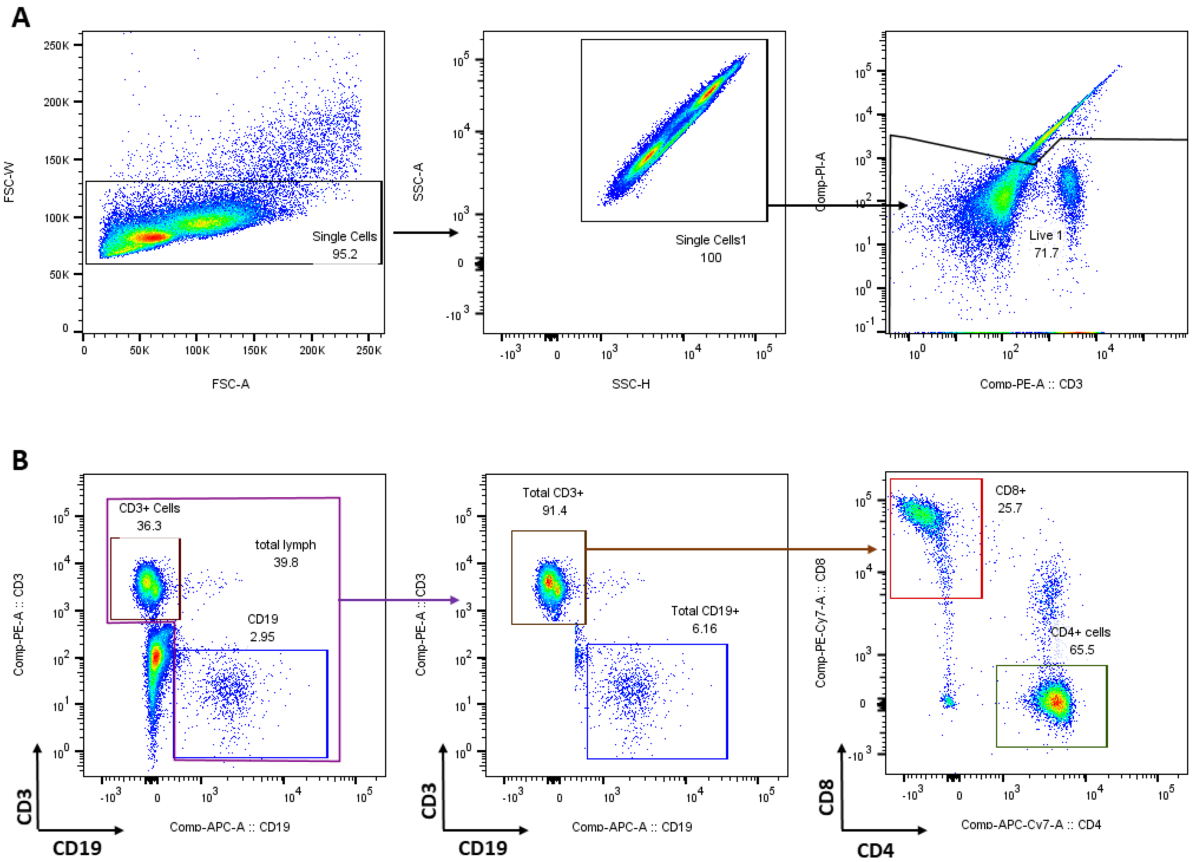

Figure S6. Gating strategy for Regulatory T cells (Panel C). (**A**) Doublets were excluded from the analysis using side scatter Height (FSC-W) and side scatter area (FSC-A), followed by live cells. (**B**) The expression of T cells was identified by CD3+ cells. Under this population CD4+ helper T cells were identified, followed by identification of CD25+CD127- cells. Within this population, regulatory T cells (Tregs) were identified using the expression of CD4+CD25+FOXP3+ cells.

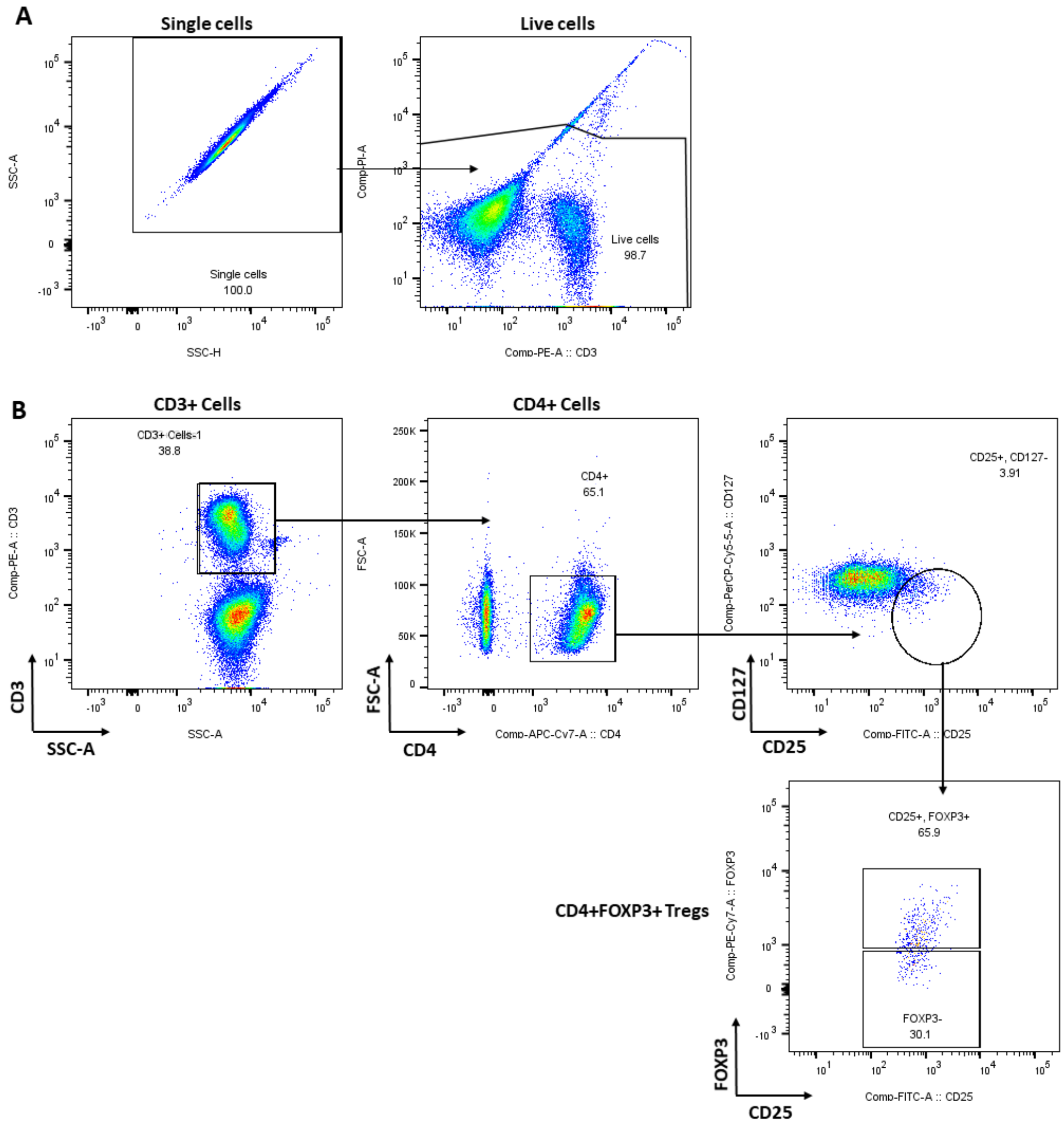

Figure S7. Gating strategy for Regulatory B cells (Panel D). (**A**) Doublets were excluded from the analysis using forward scatter width (FSC-W) and forward scatter area (FSC-A). Single cells were identified using side scatter area (SSC-A) and side scatter height (SSC-H). This followed by identification of live cells. (**B**). Using forward scatter area (FSC-A) and CD3+ cells, CD3- cells were gated (Red gate), and B cells were

identified by expression of CD19+ cells (Blue gate). Within CD19+ cells, Regulatory B cells were identified by expression of $CD38^{hi}CD24^{hi}$ cells (Red gate).

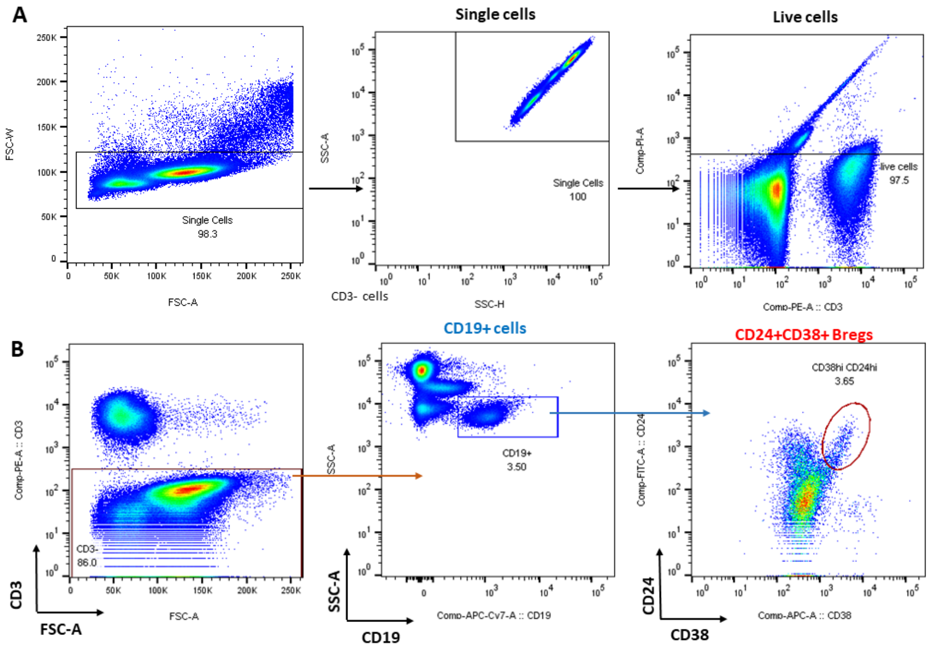

Figure S8. Gating strategy for the activated monocytes (Panel E). (A) Doublets were excluded from the analysis using forward scatter width (FSC-W) and forward scatter area (FSC-A), followed by

identification of single cells and the Live cells. (**B**). By excluding T, B, and NK cells, monocytes were gated (Violet gate) by SSC-AhiCD16lo cells. Within the monocytes gate (Violet gate), activated monocytes were identified by the expression of CD80+ (Green gate), CD86+ (Blue gate), and CD80+CD86+ cells (Red gate).

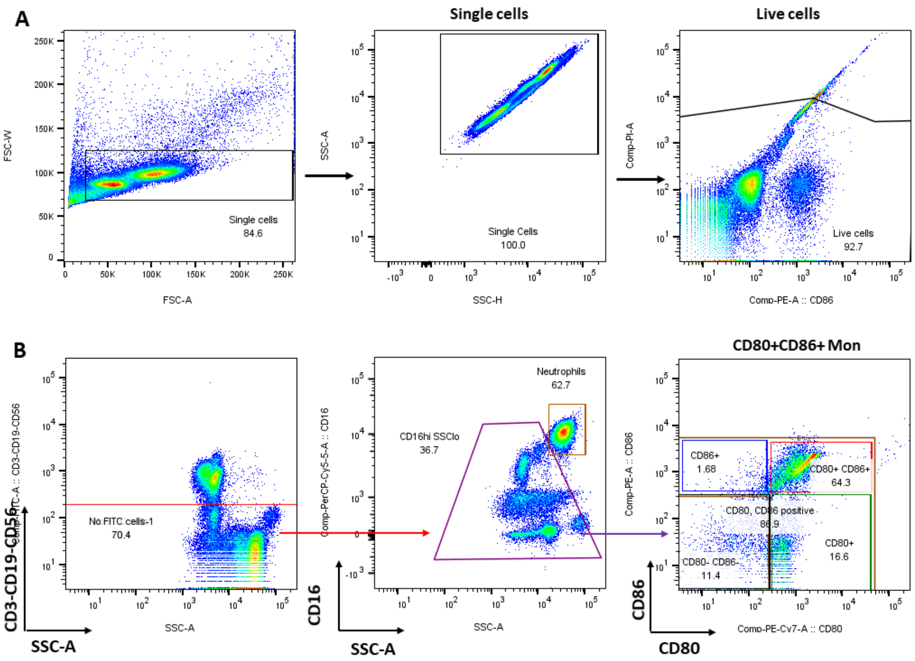

Supplementary Table

Table S1. Antibody characteristics used for immune cell phenotyping.

Anti-human	Clone	Fluorophore	Cat. No	Supplier
**CD3**	UCHT1	PE	555333	BD
**CD3**	UCHT1	FITC	50-936-9	eBioscience™
**CD4**	SK3	PE-Cy7	557852	BD
**CD4**	SK3	APC-Cy7	341095	BD
**CD8**	SK1	PE-Cy7	335787	BD
**CD14**	MφP9	Alexa Flour 647	562690	BD
**CD16**	3G8	PerCP-Cy™5.5	560717	BD
**CD19**	SJ25C1	APC-Cy™7	557791	BD
**CD19**	HIB19	APC	555415	BD
**CD56**	NCAM16.2	FITC	340410	BD
**CD25**	2A3	BB515/FITC	564467	BD
**CD127**	HIL-7R-M21	PerCP-Cy™5.5	560551	BD
**CD24**	ML5	FITC	560992	BD
**CD38**	HIT2	APC	560980	BD
**CD19**	SJ25-C1	FITC	OB9340-02	Southern Biotech™
**CD80**	L307.4	PE-Cy7	561135	BD
**CD86**	IT2.2	PE	12-0869-42	eBioscience™
**FOXP3**	PCH101	PE-Cy7	50-245-746	eBioscience™

Additional information

1) <u>Author Study Roles</u>

Asghar Abbasi	Study Conception & Design, Subject Recruitment, Training, Da Collection & Analysis, Immunological Assessments and Analysi Manuscript Preparation and Editing.
Chiara Gattoni	Data Collection, Statistics, and Manuscript Editing
Michelina Iacovino	Flow Cytometry and Manuscript Editing
Jacqueline Tosolini	Exercise Pulmonary Rehabilitation and Subject Education
Carrie Ferguson	Data Collection, Manuscript Editing and Review
Janos Porszasz	Manuscript Review and Editing
Ashrita Singh	Data Entry & Review, Quality Checks, and Manuscript Review
Kyaw Soe	Data Entry and Manuscript Review
Charles Lanks	Subject Recruitment, Manuscript Review
Harry Rossiter	Study Conception & Design, Manuscript Review and Editing
Richard Casaburi	Study Conception & Design, Manuscript Review and Editing
William Stringer	Study Conception & Design, Subject Recruitment, Training, Subject Education, Data Collection & Analysis, Manuscript Preparation, Manuscript Editing, Final Responsibility for Data Integrity

2) <u>Acknowledgement Study Roles</u>

Leticia Diaz	Study Coordinator
Robert Calmelat	Exercise Physiologist
Agustin Leyva	Study Coordinator

3) An abstract describing some partial results of this study was presented at the 2023 European Respiratory Society Meeting in Milan, Italy, abstract # 1187.

4) A paper describing the lack of change in important CPET variables with the two day CPET protocol is currently under review at another journal. Therefore, a portion of the pre-exercise training CPET data from this manuscript may be published subsequently. The post training CPET data, PROs and immunology data in this manuscript have not been published before.

Supplementary References

1. Ware, J.E., Jr.; Sherbourne, C.D. The MOS 36-item short-form health survey (SF-36). I. Conceptual framework and item selection. *Med. Care* **1992**, *30*, 473–483.
2. Norman, G.R.; Sloan, J.A.; Wyrwich, K.W. Interpretation of changes in health-related quality of life: the remarkable universality of half a standard deviation. *Med. Care* **2003**, *41*, 582–592. https://doi.org/10.1097/01.MLR.0000062554.74615.4C.
3. Wyrwich, K.W.; Nelson, H.S.; Tierney, W.M.; Babu, A.N.; Kroenke, K.; Wolinsky, F.D. Clinically important differences in health-related quality of life for patients with asthma: an expert consensus panel report. *Ann. Allergy Asthma Immunol.* **2003**, *91*, 148–153. https://doi.org/10.1016/s1081-1206(10)62169-2.
4. Clark, L.V.; Pesola, F.; Thomas, J.M.; Vergara-Williamson, M.; Beynon, M.; White, P.D. Guided graded exercise self-help plus specialist medical care versus specialist medical care alone for chronic fatigue syndrome (GETSET): a pragmatic randomised controlled trial. *Lancet* **2017**, *390*, 363–373. https://doi.org/10.1016/s0140-6736(16)32589-2.
5. Wyrwich, K.W.; Fihn, S.D.; Tierney, W.M.; Kroenke, K.; Babu, A.N.; Wolinsky, F.D. Clinically important changes in health-related quality of life for patients with chronic obstructive pulmonary disease: an expert consensus panel report. *J. Gen. Intern. Med.* **2003**, *18*, 196–202. https://doi.org/10.1046/j.1525-1497.2003.20203.x.
6. Krupp, L.B.; LaRocca, N.G.; Muir-Nash, J.; Steinberg, A.D. The fatigue severity scale. Application to patients with multiple sclerosis and systemic lupus erythematosus. *Arch. Neurol.* **1989**, *46*, 1121–1123. https://doi.org/10.1001/archneur.1989.00520460115022.
7. Nordin, Å.; Taft, C.; Lundgren-Nilsson, Å.; Dencker, A. Minimal important differences for fatigue patient reported outcome measures-a systematic review. *BMC Med. Res. Methodol.* **2016**, *16*, 62. https://doi.org/10.1186/s12874-016-0167-6.
8. Kroenke, K.; Spitzer, R.L.; Williams, J.B. The PHQ-9: validity of a brief depression severity measure. *J. Gen. Intern. Med.* **2001**, *16*, 606–613. https://doi.org/10.1046/j.1525-1497.2001.016009606.x.
9. Löwe, B.; Unützer, J.; Callahan, C.M.; Perkins, A.J.; Kroenke, K. Monitoring depression treatment outcomes with the patient health questionnaire-9. *Med. Care* **2004**, *42*, 1194–1201. https://doi.org/10.1097/00005650-200412000-00006.
10. Spitzer, R.L.; Kroenke, K.; Williams, J.B.; Löwe, B. A brief measure for assessing generalized anxiety disorder: the GAD-7. *Arch. Intern. Med.* **2006**, *166*, 1092–1097. https://doi.org/10.1001/archinte.166.10.1092.
11. Toussaint, A.; Hüsing, P.; Gumz, A.; Wingenfeld, K.; Härter, M.; Schramm, E.; Löwe, B. Sensitivity to change and minimal clinically important difference of the 7-item Generalized Anxiety Disorder

Questionnaire (GAD-7). *J. Affect. Disord.* **2020**, *265*, 395–401. https://doi.org/https://doi.org/10.1016/j.jad.2020.01.032.
12. Mahler, D.A.; Wells, C.K. Evaluation of clinical methods for rating dyspnea. *Chest* **1988**, *93*, 580–586. https://doi.org/10.1378/chest.93.3.580.
13. Ana Luisa Araújo, O.; Lília, A.; Alda, M. Minimal clinically important difference and predictive validity of the mMRC and mBorg in acute exacerbations of COPD. *Eur. Respir. J.* **2017**, *50*, PA4705. https://doi.org/10.1183/1393003.congress-2017.PA4705.
14. Anelise Bauer, M.; Aline Almeida, G.; Juliana, A.; Júlia, Z.; Luiza Minato, S.; Manuela, K.; Anamaria Fleig, M. Modified Medical Research Council and COPD Assessment Test Cutoff Points. *Respir. Care* **2021**, *66*, 1876. https://doi.org/10.4187/respcare.08889.
15. Folstein, M.F.; Folstein, S.E.; McHugh, P.R. "Mini-mental state". A practical method for grading the cognitive state of patients for the clinician. *J. Psychiatr. Res.* **1975**, *12*, 189–198. https://doi.org/10.1016/0022-3956(75)90026-6.
16. Crum, R.M.; Anthony, J.C.; Bassett, S.S.; Folstein, M.F. Population-based norms for the Mini-Mental State Examination by age and educational level. *JAMA* **1993**, *269*, 2386–2391.
17. Watt, J.A.; Veroniki, A.A.; Tricco, A.C.; Straus, S.E. Using a distribution-based approach and systematic review methods to derive minimum clinically important differences. *BMC Med. Res. Methodol.* **2021**, *21*, 41. https://doi.org/10.1186/s12874-021-01228-7.
18. Frederikus, A.K.; Gudula, J.A.M.B.; Stefano, B.; Matthias, E.; Geelhoed, J.J.M.; Samuel, K.; Spencer, A.R.; Martijn, A.S.; Jörg, V.; Bob, S. The Post-COVID-19 Functional Status scale: a tool to measure functional status over time after COVID-19. *Eur. Respir. J.* **2020**, *56*, 2001494. https://doi.org/10.1183/13993003.01494-2020.
19. Buysse, D.J.; Reynolds, C.F., 3rd; Monk, T.H.; Berman, S.R.; Kupfer, D.J. The Pittsburgh Sleep Quality Index: a new instrument for psychiatric practice and research. *Psychiatry Res.* **1989**, *28*, 193–213. https://doi.org/10.1016/0165-1781(89)90047-4.
20. Longo, U.G.; Berton, A.; De Salvatore, S.; Piergentili, I.; Casciani, E.; Faldetta, A.; De Marinis, M.G.; Denaro, V. Minimal Clinically Important Difference and Patient Acceptable Symptom State for the Pittsburgh Sleep Quality Index in Patients Who Underwent Rotator Cuff Tear Repair. *Int. J. Environ. Res. Public Health* **2021**, *18*. https://doi.org/10.3390/ijerph18168666.
21. Cotler, J.; Holtzman, C.; Dudun, C.; Jason, L.A. A Brief Questionnaire to Assess Post-Exertional Malaise. *Diagnostics* **2018**, *8*. https://doi.org/10.3390/diagnostics8030066.
22. Hasan, M.; Beitz, B.; Rouilly, V.; Libri, V.; Urrutia, A.; Duffy, D.; Cassard, L.; Di Santo, J.P.; Mottez, E.; Quintana-Murci, L.; et al. Semi-automated and standardized cytometric procedures for multi-panel and multi-parametric whole blood immunophenotyping. *Clin. Immunol.* **2015**, *157*, 261–276. https://doi.org/10.1016/j.clim.2014.12.008.
